# Anesthesia management of patients undergoing off-pump coronary artery bypass grafting: A retrospective study of single center

**DOI:** 10.3389/fsurg.2022.1067750

**Published:** 2023-01-30

**Authors:** Yong Lin, Tao Sun, Ning-ning Cheng, Jing-jing Liu, Li-xian He, Li-hong Wang, Xian-wen Liu, Mei-fang Chen, Liang-wan Chen, Yun-tai Yao, the Evidence in Cardiovascular Anesthesia (EICA) Group

**Affiliations:** ^1^ Department of Anesthesiology, Fuwai Hospital, National Center for Cardiovascular Diseases, Peking Union Medical College and Chinese Academy of Medical Sciences, Beijing, China; ^2^ Department of Cardiovascular Surgery, Fujian Medical University Union Hospital, Fuzhou, China; ^3^ Department of Anesthesiology, The Affiliated Hospital of Inner Mongolia Medical University, Huhhot, China; ^4^ Department of Anesthesiology, Binzhou People's Hospital, Binzhou, China; ^5^ Department of Anesthesiology, The First Affiliated Hospital of Xinxiang Medical College, Xinxiang, China; ^6^ Department of Anesthesiology, Chuiyangliu Hospital of Tsinghua University, Beijing, China; ^7^ Department of Anesthesiology, Liaocheng People's Hospital, Liaocheng, China

**Keywords:** coronary artery bypass, anesthesia management, single center, retrospective, hemodynamics

## Abstract

**Background:**

To summarize the current practice of anesthesia management for Chinese patients undergoing off-pump coronary artery bypass (OPCAB) surgery at a large-volume cardiovascular center.

**Materials and methods:**

The clinical data of consecutive patients undergoing isolated, primary OPCAB surgery during the period from September 2019 to December 2019 were retrospectively analyzed. Patient characteristics, intraoperative data, and short-term outcomes were extracted from the Hospital Information System and the Anesthesia Information Management System.

**Results:**

A total of 255 patients who underwent OPCAB surgery were enrolled in the current study. High-dose opioids and short-acting sedatives were the most commonly administrated anesthetics intraoperatively. Pulmonary arterial catheter insertion is frequently performed in patients with serious coronary heart disease. Goal-directed fluid therapy, a restricted transfusion strategy, and perioperative blood management were routinely used. Rational usages of inotropic and vasoactive agents facilitate hemodynamic stability during the coronary anastomosis procedure. Four patients underwent re-exploration for bleeding, but no death was observed.

**Conclusions:**

The study introduced the current practice of anesthesia management at the large-volume cardiovascular center, and the short-term outcomes indicated the efficacy and safety of the practice in OPCAB surgery.

## Introduction

Fuwai Hospital serves as the national center for cardiovascular diseases of China and is one of the largest cardiovascular centers in the world (1). The annual cardiovascular surgical volume at Fuwai Hospital surpassed 10,000 in 2011 and has increased significantly over the last 10 years. It was reported in the 2019 annual surgery outcome report that the cardiovascular surgical volume of Fuwai Hospital was 14,808, and the 30-day postoperative mortality rate was 0.4%, which had been below 1.0% for 11 consecutive years. For patients with coronary artery diseases who underwent surgery, the 30-day postoperative mortality rate was 0.2% (8/4,247) (2). Off-pump coronary artery bypass surgery (OPCAB) was first reported by Saint Petersburg (3) in 1964 and became prevalent around the world in the 1990s owing to the invention of the anastomosis site restraining device (“Octopus”) by Borst et al. (4). Cardiovascular anesthesia is risky, which is full of challenge and debate, especially during the OPCAB surgery. Controversies never stop in terms of hemodynamic monitoring, anesthesia maintenance, anesthetic selection, infusion strategy, anticoagulation and reversal, perioperative blood management, and so on. The Anesthesia Department at Fuwai Hospital has been improving and developing continuously. The aim of this study was to present the current anesthesia practice in isolated OPCAB surgery at Fuwai Hospital.

## Materials and methods

### Study design and patient population

During the period from September 2019 to December 2019, consecutive adult (≥18 years) patients scheduled for isolated OPCAB surgery at Fuwai Hospital were enrolled in this retrospective study. Patients who underwent any additional cardiovascular surgery or the conventional coronary artery bypass surgery under cardiopulmonary bypass, as well as those who underwent cardiac surgery previously or were younger than 18 years old, were excluded from this study. Patient characteristics (e.g., age, gender, body mass index, comorbidities, coronary lesion, and cardiac function), intraoperative data (e.g., surgical duration, coronary graft number and patency, cardiopulmonary bypass conversion, hemodynamic monitoring, anesthetics, anticoagulation and reversal, inotropic and vasoactive agents, fluid infusion, and blood transfusion), and postoperative outcomes [e.g., mortality, mechanical ventilation duration, length of stay in the intensive care unit (ICU) and hospital and complications] were extracted from the Hospital Information System (HIS) and the Anesthesia Information Management System (AIMS).

### Surgical protocol

All of the OPCAB surgeries were performed by the chief surgeons. All patients were operated on through a full median sternotomy in the supine position. The left internal mammary artery was harvested routinely. When required, a tract of the great saphenous vein or radial artery was also harvested through a standard open approach. An initial 200 IU kg^−1^ heparin and repeat intravenous injections were administered to obtain an activated clotting time (ACT) ≥300 s. After revascularization, a quantitative bypass blood flow measurement using an ultrasonic transit-time flow meter (MiraQ™, Medistim, Oslo, Norway) was then utilized for the evaluation of graft patency. The measured flow should be pulsatile and biphasic, with two forward components of short presystolic and large diastolic flow, and one small component of retrograde telesystolic flow. The mean graft flow is at least 15 ml min^–1^, and the optimal pulsatility index (PI) is between 1 and 5 (5). After the confirmation of graft patency, the effect of heparin was reversed by protamine with a ratio of 0.5–0.8:1 (1:1 was defined as 1 mg protamine per 100 IU heparin), which depended on the latest ACT value, cumulative dosage of heparin, and duration of graft anastomosis.

### Anesthesia management

All patients received general anesthesia, and they were kept normothermic. The peripheral artery and the central venous catheters were routinely performed for invasive hemodynamic monitoring. Intraoperative administration was performed *via* peripheral pathway (induction and fluid infusion) or central venous catheter (maintenance). Core temperature was monitored *via* bladder catheter. Five-lead electrocardiogram was routinely applied for myocardial ischemia and malignant arrhythmia. When necessary, transesophageal echocardiography (TEE) was performed to evaluate ventricular wall motion abnormalities, intracardiac thrombi, ventricular aneurysm, valve inefficiency, and volume status. The right femoral artery site was catheterized for an intra-aortic balloon pump, and the pulmonary arterial catheter (PAC) was placed in patients with seriously impaired cardiac function or with pulmonary arterial hypertension.

Mean arterial pressure was kept (MAP)/heart rate (HR) ≥1.0 and MAP ≥70 mmHg throughout the surgery. A reduced cardiac output was accepted as long as the mixed venous oxygen saturation (SvO_2_) remained >60% and metabolic acidosis did not develop. Elevating the legs, utilizing the vasoconstrictors, increasing the fluid administration, and maintaining an optimal HR ranging from 50 to 70 beats per minute were performed when hypotension occurred. The surgery was converted to on-pump with the persistence of the following settings for >15 min despite aggressive therapy (5): (1) Cardiac index <1.5 L min m^−2^; (2) SvO_2 _< 60%; (3) MAP < 50 mmHg; (4) ST segment elevation >2 mV; (5) Large new wall motion abnormalities or collapse of left ventricular function assessed by TEE; (6) Sustained malignant arrhythmias.

The threshold for transfusion of allogeneic red blood cells was a hemoglobin concentration ≤9 g/dl or a hematocrit value ≤27%. The criteria for a fresh frozen plasma transfusion were a prothrombin time 1.5 times longer than baseline with diffuse bleeding. The protocol for platelet transfusion was the presence of diffuse bleeding and a platelet count <50,000/mm^3^ or platelet dysfunction. All patients underwent intraoperative cell salvage with autotransfusion of washed salvaged red cells at the end of the operation.

### Statistical methods

All statistical analyses were performed using SPSS for mac (25.0, IBM, United States). The continuous variables were tested for normality first and then expressed as mean ± standard deviation (normal distributions) or median and interquartile range (nonnormal distributions), and the categorical variables were presented as numbers and percentages. The histogram and line chart were utilized in the representation of the numerical data *via* GraphPad Prism (8.0, GraphPad Software, United States) and Office (2010, Microsoft, United States).

## Results

### Baseline variables and short-term outcomes

Between September 2019 and December 2019, a total of 255 patients were enrolled in this sampling survey who conformed to the screening criteria. All details of the baseline demographic and clinical characteristics are presented in Table 1. The majority of the procedures were planned to include three or more than three coronary grafts (45.1% and 33.3%, respectively). The mean graft flow and PI are displayed in Table 2. Few patients received blood product transfusion (2.4%) or re-exploration for bleeding (1.6%). No death was observed in the hospital after the OPCAB surgery (Table 2).

**Table 1:** Baseline characteristics.

Baseline demographics	Data (*n* = 255)
Age, years	62.0 (55.0–67.0)
Male, *n* (%)	190 (74.5)
Body mass index	25 (23.8–27.0)
Emergency, *n* (%)	3 (1.2)
Active smoker	129 (50.6)
Alcohol abuse, *n* (%)	34 (13.3)
**Baseline cardiac function, *n* (%)**	
NYHA ≥ class III	64 (25.1)
More than three impaired coronary vessels	85 (33.3)
Prior percutaneous coronary intervention	43 (16.9)
Prior myocardial infarction	
≥3 months	54 (21.2)
Within 3 months	18 (7.1)
Angina	218 (85.5)
Arrhythmia	11 (4.3)
Left ventricular ejection fraction	
≥50%	251 (98.4)
30–50%	4 (1.6)
<30%	0 (0.0)
**Comorbidities, *n* (%)**	
Hypertension	173 (67.8)
Diabetes	87 (34.1)
Stroke	24 (9.4)
Peripheral artery stenosis	16(6.3)
Chronic pulmonary disease	9 (3.5)
Hyperlipidemia	194 (76.1)
Renal insufficiency^a^	3 (1.2)

The categorical variables were presented as *n*, (%), and the distributions variables were presented as mean ± standard deviation (normal distributions) or median and interquartile range (nonnormal distributions).

NYHA, New York Heart Association.

^*a*^ Serum creatinine >200 μmol/L or requiring dialysis.

**Table 2:** Surgical characteristics and short-term outcomes.

Category	Data (*n* = 255)
**Coronary grafts, *n* (%)**	
One	13 (5.1)
Two	41 (16.1)
Three	115 (45.1)
More than three	85 (33.3)
Mean	3.0 (3.0–4.0)
**Graft materials, *n***	
Arterial	1.0 (1.0–1.0)
Venous	2.0 (1.0–3.0)
**Graft flow, L min^−1^/pulsatility index**	
Anterior descending artery	26.0 (18.0–39.2)/2.1 (1.8–2.7)
Right coronary artery	37.0 (21.0–50.0)/1.6 (1.2–2.2)
Left circumflex branch	35.0 (23.0–70.0)/2.0 (1.6–2.7)
Obtuse marginal branch	36.0 (25.0–52.0)/2.0 (1.5–2.3)
Posterior descending branch	34.0 (25.0–49.8)/1.9 (1.3–2.4)
Diagonal branch	36.0 (23.5–55)/2.0 (1.5–2.3)
Surgery time, min	211.0 (175.0–245.8)
Hemorrhage, ml	581.0 ± 102.6
Any blood consumption, *n* (%)	6 (2.4)
Hemostatic administration, *n* (%)	8 (3.1)
Urine output, ml	500.0 (300.0–800.0)
**Fluid infusion**	
Total, ml	1200.0 (800.0–1500.0)
Crystalloid, ml	700.0 (500.0–1,000)
Colloid, ml	300.0 (0.0–500.0)
Cry/col	1.6 (1.0–2.5)
**Outcomes in hospital**	
All-cause death, *n* (%)	0 (0.0)
Mechanical ventilation duration, days	1.1 ± 0.5
Length of stay in intensive care unit, days	2.0 (1.0–4.0)
Length of stay in hospital after surgery, days	7.0 (7.0–8.0)
Re-exploration for bleeding, *n* (%)	4 (1.6)
Red blood cell transfusion, ml	0.8 ± 2.0
Thoracic drainage, ml	875.0 (687.5–1200.0)
Hemoglobin, g/L	116.0 (101.0–126.0)

The categorical variables were presented as *n* (%), and the distributions variables were presented as mean ± standard deviation (normal distributions) or median and interquartile range (nonnormal distributions).

### Anesthetic management

**Figure 1:**
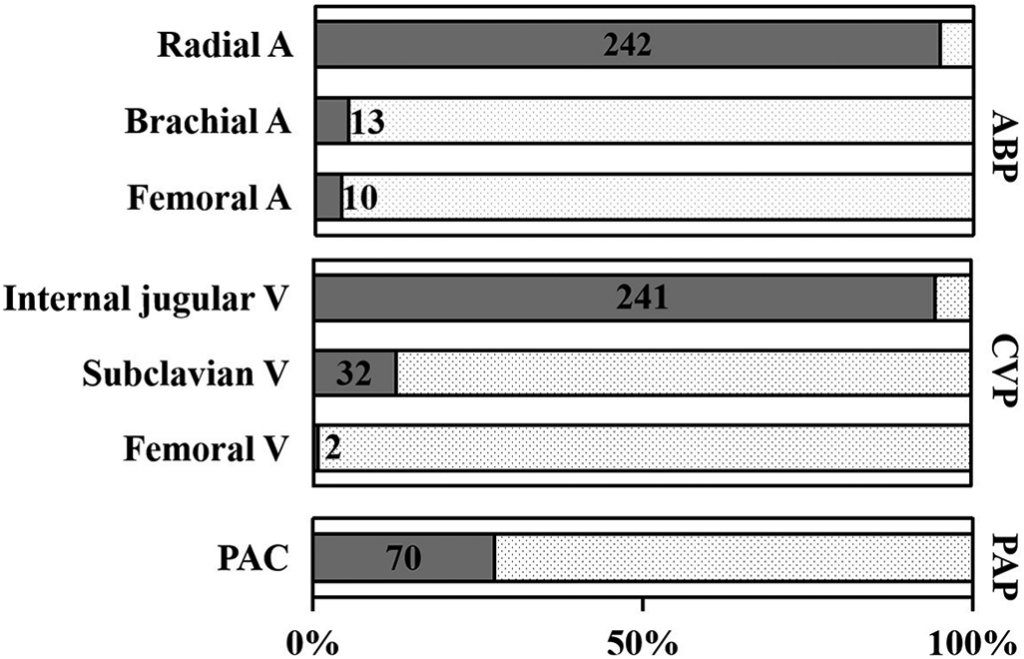
Hemodynamic monitoring. Radial A was the most conventional arterial blood pressure monitoring site during the operation (242/255, 94.9%). Femoral A puncture (10/255, 5.1%) was applied in the postoperative placement of the intra-aortic balloon pump (IABP) or alternative site while difficult catheterization of Radial A or Brachial A occurred. Internal V (241/255, 94.5%) was the most conventional punctual site in central venous puncture, and PAC (70/255, 27.5%) was utilized in less than one third of OPCAB patients. ABP, arterial blood pressure; CVP, central venous pressure; A, artery; V, vein; PAP, pulmonary arterial pressure; OPCAB, off-pump coronary artery bypass; PAC, pulmonary arterial catheter.

Chief or associate chief anesthesiologists participated in 63.5% (162/255) of the OPCAB surgeries. The radial artery was chosen as the most common site for invasive arterial blood pressure monitoring (242/255, 94.9%) and the brachial artery, dorsal pedal artery, or femoral artery were alternatives for invasive arterial blood pressure monitoring when puncture difficulty occurred. Central venous catheter was performed *via* right internal jugular vein in most cases (241/255, 94.5%). PAC was applied to one-fourth of the patients (Figure 1).

**Figure 2:**
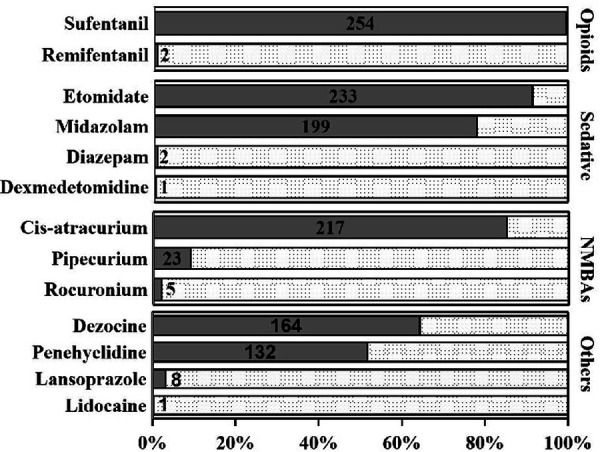
Frequency of anesthesia agents utilization during anesthesia induction period. Sufentanil (254/255, 99.6%), etomidate (233/255, 91.4%), and cisatracurium (217/255, 85.1%) were the most frequent anesthesia agents during induction period.

**Figure 3:**
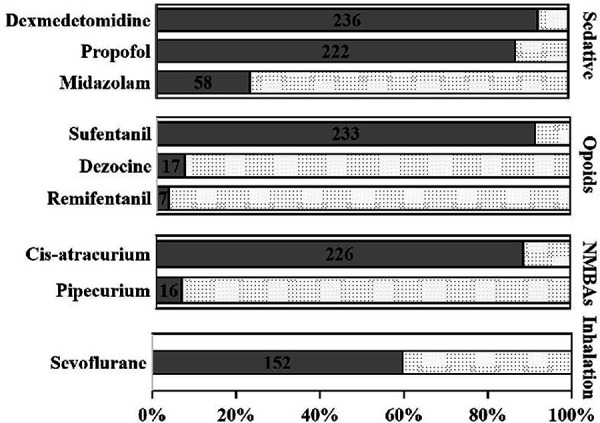
Frequency of anesthesia agents application during anesthesia maintenance period. Dexmedetomidine (236/255, 92.5%), sufentanil (233/255, 91.4%), cisatracurium (226/255, 88.6%), and propofol (222/255, 87.1%) were the most frequent anesthesia agents during maintenance period.

None of the patients received anesthetic premedication. Sufentanil (254/255, 99.6%), etomidate (233/255, 91.4%), and cisatracurium (217/255, 85.1%) were the most frequent anesthesia agents the during induction period. During the induction and maintenance periods, 150.0 (100.0, 200.0) μg and 175 (100.0, 225.0) μg sufentanil were utilized, and the total dosage was 300.0 (255.0, 350.0) μg. Remifentanil was adiministered in very few patients (7/255, 2.7%) during anesthesia maintenance, and the mean dosage was 450.0 (305.0, 1640.0) μg. Dexmedetomidine, sufentanil, cisatracurium, and propofol were regularly utilized during anesthetic maintenance in most cases (92.5%, 91.4%, 88.6%, and 87.1%, respectively) (Figures 2, 3).

**Figure 4:**
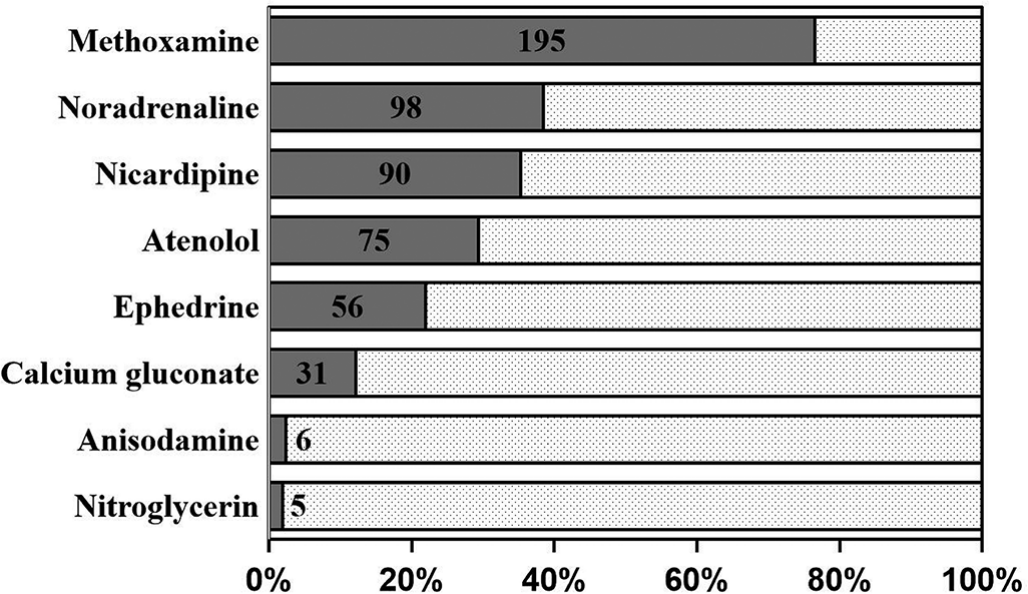
Inotropic and vasoactive agents (bonus). Methoxamine (195/255, 76.5%) and norepinephrine (98/255, 38.4%) were the most common vasoconstrictors with a fashion of bonus when hypotension occurred.

**Figure 5:**
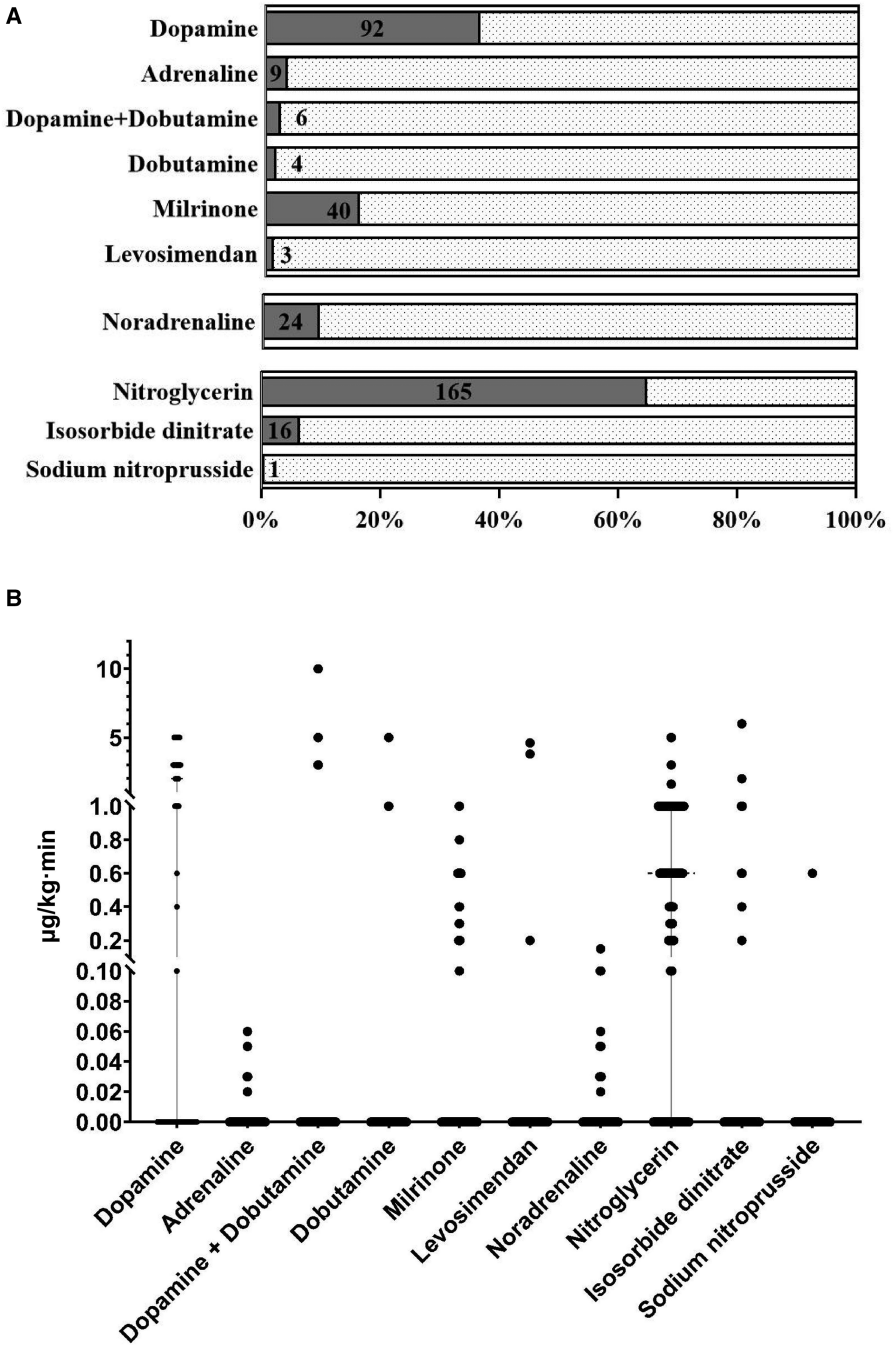
(**A**) The frequencies of the inotropic and vasoactive agents usages (continuous infusion) at the end of the surgery. Nitroglycerin (165/255, 64.7%) and dopamine (92/255, 36.1%) were the most frequent vasoactive agents with a fashion of continuous infusion. (**B**) The individual dosages of inotropic and vasoactive agents were displayed in the scatter plot.

**Figure 6:**
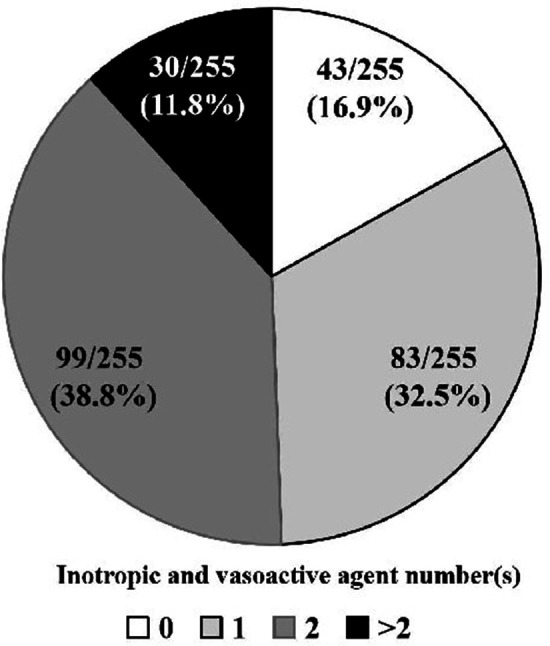
Administration of inotropic and vasoactive agents at the end of the operation. Almost all the patients were sent to ICU with at least one kind of inotropic or vasoactive agent (212/255, 83.1%). ICU, intensive care unit.

**Figure 7:**
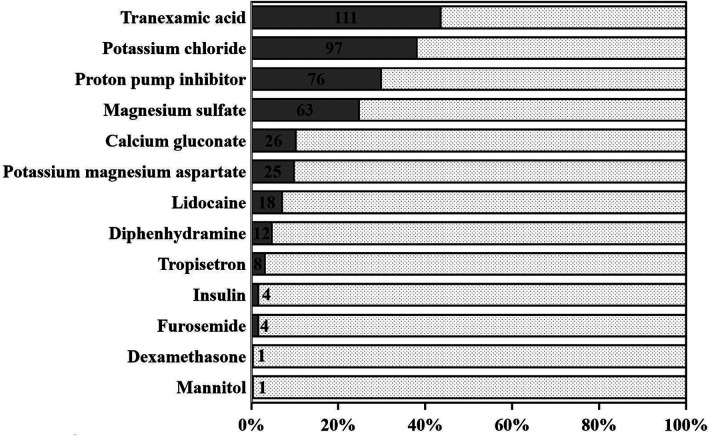
Adjuvant drugs. Tranexamic acid (111/255, 43.5%), potassium chloride (97/255, 38.0%), and proton pump inhibitor (76/255, 29.8%) were the most frequent adjuvant drugs.

Methoxamine (195/255, 76.5%) and norepinephrine (98/255, 38.4%) were the most common vasoconstrictors, with a fashion of bolus when hypotension occurred (Figure 4). The majority of the patients were sent to the ICU with at least one kind of inotropic or vasoactive agent (211/255, 83.1%), of which nitroglycerin (165/255, 64.7%) and dopamine (92/255, 36.1%) were applied frequently (Figures 5, 6). Antifibrinolytic agent tranexamic acid (TXA) was applied to 43.5% of the patients, and the dosage was 3.0 (3.0, 3.0) g. Potassium chloride was usually used to correct hypokalemia and prevent arrhythmia (97/255, 38.0%) (Figure 7).

**Figure 8:**
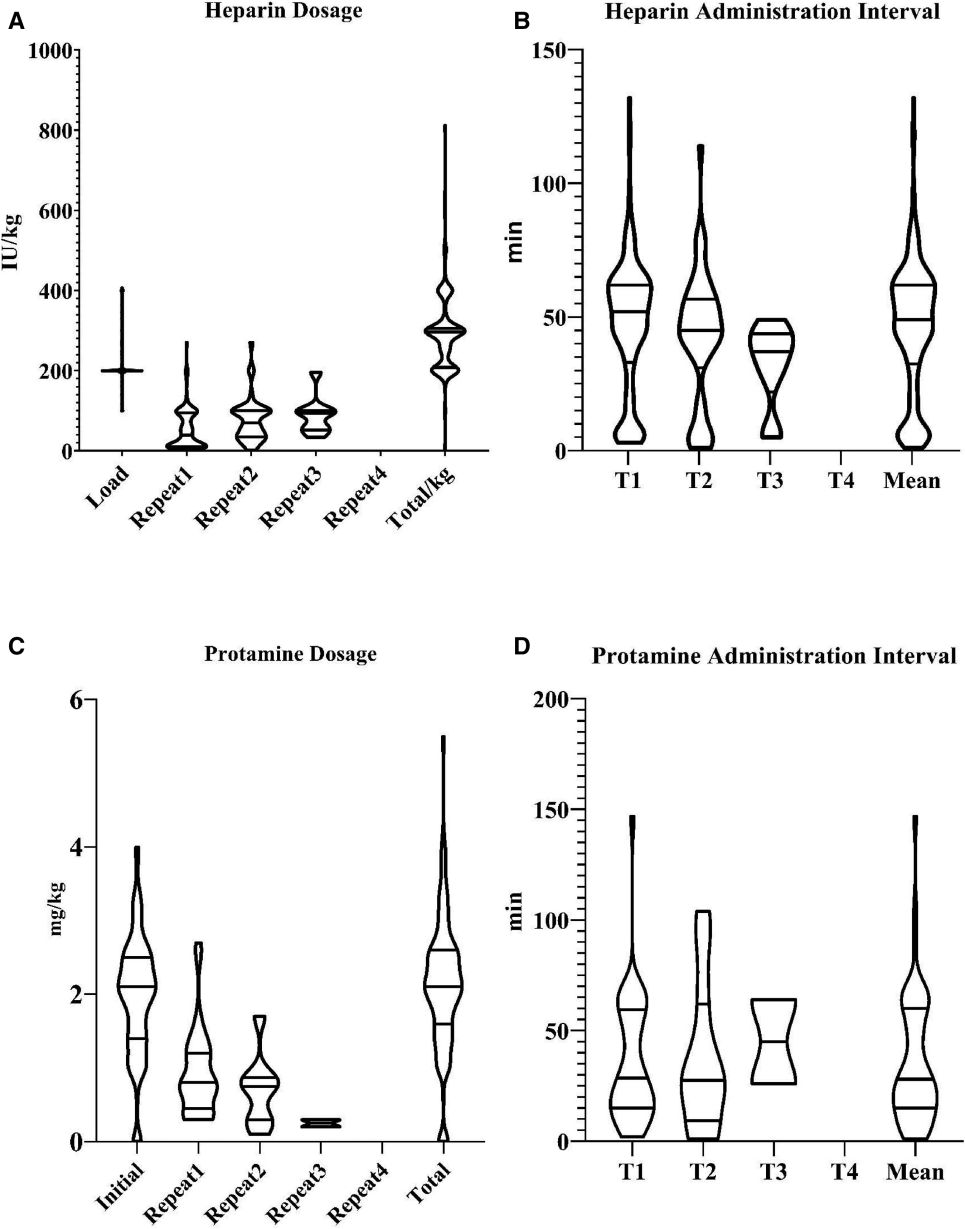
Heparinization and reversal strategy. Violin plot was applied to display the intraoperative anticoagulation and reversal strategy. The width of the “violin” indicated the distribution and the probability density of the data. The lines inside the “violin” indicated the median and the interquartile range. T1: Interval between the loading injection and the first repeated injection; T2: Interval between the second and third repeated injection; T3 and T4 followed the similar fashion.

Total of 20,000.0 (1,600.0, 2,400.0) IU heparin was delivered during the surgery, and the repeating times throughout the surgical procedure were 1.0 (0.0, 1.0). Heparinization and reversal strategies are demonstrated in Figure 8. Allogeneic transfusion was performed in only 2.4% (6/255) of our series, and a minority of patients received hemostatic administration (hemocoagulase agkistrodon) (3.1%, 8/255) (Table 2). Blood gas analyze results are displayed in Table 3.

**Table 3:** Blood gas analysis.

	Venous blood		Arterial blood	
	Pre-OP (*n* = 44)	Post-OP (*n* = 13)	Pre-OP (*n* = 191)	Post-OP (*n* = 125)
Partial pressure of oxygen, mmHg	41.3 (38.2 to 45.4)	43.8 (40.1 to 48.5)	225.6 (157.8 to 295.4)	191.2 (117.1 to 262.2)
Partial pressure of carbon dioxide_,_ mmHg	42.9 (39.5 to 45.6)	43.4 (40.8 to 47.6)	34.9 (32.6 to 38.9)	35.1 (31.3 to 39.4)
Chloridion, mmol/L	107.6 (106.2 to 109.1)	109.1 (107.4 to 111.2)	110.1 (108.6 to 111.7)	112.7 (109.3 to 115.1)
Potential of hydrogen	7.4 (7.4 to 7.4)	7.4 (7.3 to 7.4)	7.4 (7.4 to 7.4)	7.4 (7.4 to 7.4)
Calcium ion, mmol/L	1.2 (1.2 to 1.3)	1.3 (1.2 to 1.3)	1.3 (1.2 to 1.3)	1.2 (1.2 to 1.3)
Potassium ion, mmol/L	4.0 (3.8 to 4.3)	4.3 (4.0 to 4.6)	4.1 (3.7 to 4.6)	4.3 (4.0 to 4.6)
Sodium, mmol/L	140.5 (139.1 to 141.7)	140.3 (138.6 to 141.7)	140.6 (139.1 to 141.7)	140.8 (139.3 to 141.5)
Hematocrit, %	36.0 (34.0 to 39.0)	34.0 (31.0 to 37.0)	34.0 (31.0 to 36.0)	32.0 (27.0 to 35.8)
Bicarbonate radical, mmol/L	25.1 (24.0 to 26.2)	23.8 (22.4 to 24.7)	23.4 (21.4 to 24.9)	23.2 (21.3 to 24.1)
Glucose, mmol/L	6.2 (5.4 to 7.7)	7.9 (6.8 to 9.6)	6.4 (5.8 to 7.6)	7.4 (6.0 to 8.0)
Lactate, mmol/L	0.9 (0.8 to 1.2)	1.0 (0.9 to 1.4)	0.7 (0.6 to 0.9)	0.8 (0.8 to 0.9)
Magnesium ion, mmol/L	0.5 (0.5 to 0.6)	0.6 (0.5 to 0.7)	0.6 (0.5 to 0.8)	0.7 (0.5 to 0.8)
Oxygen saturation, %	74.9 (69.6 to 78.2)	75.1 (69.4 to 80.1)	99.8 (99.3 to 99.9)	99.0 (95.5 to 99.9)
Hemoglobin, g/L	119.0 (110.8 to 126.3)	112.0 (103.0 to 122.8)	115.0 (103.0 to 120.0)	102.0 (89.0 to 119.0)
Blood urea nitrogen, mmol/L	11.7 (9.9 to 14.0)	12.0 (11.0 to 14.3)	11.7 (8.6 to 13.0)	9.5 (8.5 to 13.0)
Base excess in extracellular fluid, mmol/L	0.6 (−1.1 to 2.0)	−1.1 (−2.8 to 0.1)	−2.2 (−5.0 to −0.6)	−2.6 (−5.8 to −1.7)

The perioperative blood gas analysis results. The categorical variables were presented as *n*, (%), and the distributions variables were presented as mean ± standard deviation (normal distributions) or median and interquartile range (nonnormal distributions).

OP, operation.

### Vital signs during the surgery

**Figure 9:**
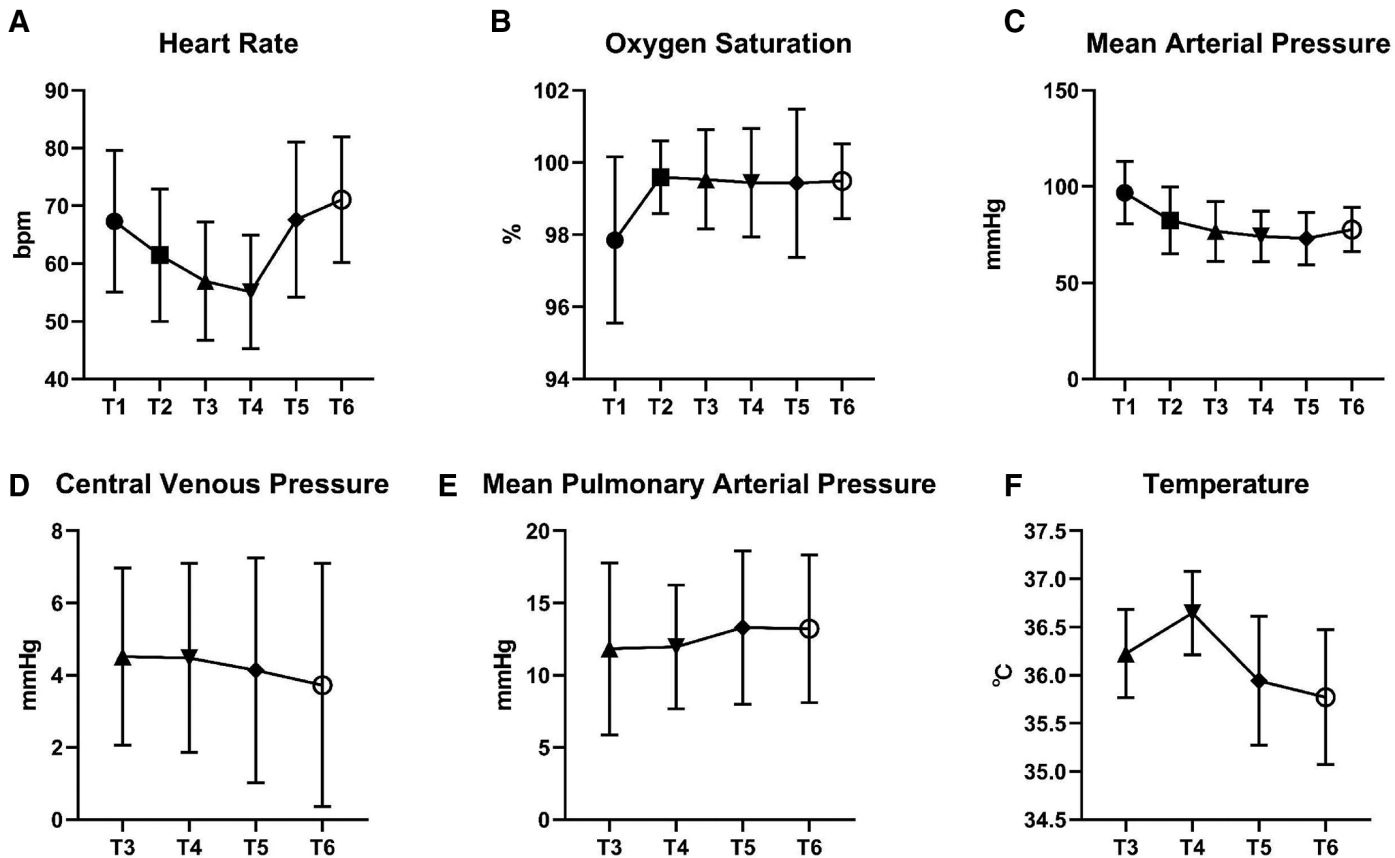
Vital signs during the surgery. The hemodynamic fluctuation, oxygenation, and core temperature during the surgery are displayed in this figure. T1 = Before induction (●); T2 = After endotracheal intubation (▪); T3 = Skin incision (▴); T4 = Protamine administration (▾); T5 = During sternum closure (◆); T6 = End of operation (○).

The lowest HR was observed after endotracheal intubation in a total of 104 patients (104/255, 40.8%). In most cases, the highest MAP happened before induction (99/255, 38.8%). The graft anastomosis period (between T3 and T4) was the most probable time point when the lowest MAP (196/255, 76.9%) and the highest HR (104/255, 40.8%) occurred. The tendencies of the hemodynamic parameters, the oxygenation, and the core temperature during the surgery are displayed in Figure 9.

## Discussion

In our series, patients were younger compared with those from Occident (6, 7). Unfortunately, the preoperative status of the patients was not optimistic in our survey. New York Heart Association (NYHA) class III or higher was observed in one-fourth of the patients. History of myocardial infarction accounted for 28.3% of the patients. Three or more impaired coronaries could be found in nearly 80% of the patients, and 16.9% of the patients experienced percutaneous coronary intervention. Therefore, graft patency and long-term survival appeared to be crucial.

### Hemodynamic monitoring

Although controversy still exists, the radial artery is the most commonly used for invasive arterial blood pressure monitoring during cardiac surgery (8). The central venous catheter *via* internal jugular vein is the initial selection at Fuwai Hospital because of its low risk of complications (9). PAC was first introduced in clinical practice in the 1970s (10), and it provides a unique and comprehensive evaluation of the cardiovascular status of patients during cardiac surgeries. In addition, it provides information on the adequacy of cardiac output by measurements of SvO_2_ and on left heart function through pulmonary artery wedge pressure and right heart function with the measurement of pulmonary arterial pressure and central venous pressure (CVP). The utilization of PAC during OPCAB surgery has been previoously demonstrated to be associated with increased mortality and a higher risk of severe end-organ complications (11). Moreover, as TTE and other types of hemodynamic monitoring such as the FloTrac/Vigileo system became popular (12), trends of PAC usage markedly decreased at the end of the 20th century (13). Interestingly, multiple studies have currently addressed that use of a PAC is associated with better outcomes after adult cardiac surgeries with low morbidity of PAC-related complications (14). After all, the reliability and integrity of hemodynamic data are the obvious advantage of PAC, especially in those patients with cardiac dysfunction. In view of this, PAC was performed on one-fourth of the cases in our series, and the complications associated with it were infrequent.

### Anesthesia maintenance

Hemodynamic abnormalities and perioperative myocardial ischemia are the most challenging events during OPCAB. The constriction of the epicardial restraining device and the temporary arrest of coronary flow are the main reasons. While the utilization of propofol for the sedation was common during the anesthesia maintenance, few patients with serious cardiovascular inhibition were observed in our series. It revealed that usage of the short-acting propofol during cardiovascular surgery might be safe, and it facilitated to accelerate the process of extubation. Excessive stress simulations during cardiac surgery, such as sternum sawing and heart positioning, lead to a significant hemodynamic response and increase myocardial oxygen assumption due to the hypertension and the tachycardia. The missions of the anesthesiologists are to suppress these adverse reactions and to maintain hemodynamic stability and tissue oxygen delivery-consumption balance. Therefore, patients in our series received a high dosage of sufentanil during the surgery, which was significantly higher than the patients who underwent noncardiac surgery. Then, they were converted into the “hibernation-like” status after induction, which was characterized by the simultaneous decline of the HR, the blood pressure, the core temperature, and the bispectral index value (40–50). Tiny response to surgical stimulation existed, and the hemodynamic stability was easily manipulated by vasoactive agents.

### Fluid management

The debate of the perioperative fluid infusion strategy in cardiac surgery still exists. All of the available surveys and real-life studies on fluid management show great variability among centers, and even among physicians at the same center (15–17). A liberal infusion strategy can reduce the incidence of tissue hypoperfusion resulting from inadequate fluid resuscitation but increase the risks of pulmonary edema and congestive heart failure (18, 19). Furthermore, the “full” cardiac chambers increase difficulty in heart positioning during coronary anastomosis.

Goal-directed fluid therapy (GDFT) was first reported by Gan et al. in 2002 (20) and was recommended by the 2019 guidelines for perioperative care in cardiac surgery to reduce postoperative complications (21). GDFT has been performed at Fuwai Hospital for several years. The individualized fluid infusion was adjusted according to the patient's volume status as judged by hemodynamic stability, fluid infusion response, blood lactate concentration, CVP, pulmonary artery wedge pressure, stroke volume variation, cardiac index, echocardiographic evaluation, and so on. It revealed that the total amount of fluid infusion during OPCAB was only 1,200 ml approximately, which was significantly less than those in major noncardiac surgeries. Although the urine output was lower than 600 ml in average, no signs of hypoperfusion were discovered during the surgery, such as hypovolemia-related hemodynamic instability, metabolic acidosis, or increased utilization of vasoactive agents.

Currently, no consensus has achieved as to which fluid is best for the patients undergoing cardiac surgery (22). The adverse effects of crystalloid fluids are usually related to their preferential distribution to specific interstitial areas (e.g., the subcutis, the gastrointestinal tract, and the lungs) (23). However, excessive infusion of colloid is a risk factor for acute renal dysfunction and coagulopathy (24). The median crystal-colloid ratio was 1.6 at Fuwai Hospital, which was similar to the ratio (1.5) reported by Cortés et al. in a meta-analysis (25). Because reported crystal-colloid ratios are quite variable, largely related to the heterogeneity of study populations and the amounts of crystalloid administered concomitantly with colloid, higher level evidence is required for further confirmation.

### Perioperative blood management

Due to perioperative blood management strategy, blood product consumption has been declining during the last decade at Fuwai Hospital (2). Few patients receive blood transfusions during cardiac surgeries. TXA has been proven to be effective in reducing postoperative bleeding and the requirement for allogeneic transfusion in patients undergoing OPCAB surgery (26). In consideration of prophylaxis of excessive hemorrhage during the surgery, antifibrinolytics was applied by 43.5% of the anesthesiologists in this study. The investigation about the association between tranexamic acid and convulsive seizures after cardiac surgery (ATCAS study) revealed that there was no evidence that TXA increased the risk of death and thrombotic complications after coronary artery surgery. A study that enrolled 11,529 patients who underwent cardiac surgery demonstrated that the incidence of postoperative seizures was 0.9% and only the TXA with a dosage exceeding 80 mg kg^−1^ was the independent risk factor for postoperative seizures (27).

The controversy between restricted and liberal allogenic transfusion concepts in cardiac surgery never stops. Mazer et al. demonstrated that a restrictive transfusion strategy in patients undergoing cardiac surgery who were at moderate-to-high risk for death was not inferior to a liberal strategy with respect to the composite outcomes of all-cause death, myocardial infarction, stroke, or new-onset renal failure with dialysis with less blood transfused (28). However, a substudy with respect to the red blood cell transfusion in elderly patients undergoing cardiac surgery (TRACS study) discovered that a restrictive transfusion strategy might result in an increased incidence of cardiogenic shock in elderly patients undergoing cardiac surgery compared with a more liberal strategy. The cardiovascular risk of anemia may be more harmful than the risk of blood transfusion in older patients (29). While there is no consensus about which transfusion strategy is feasible and safer, restricted allogenic transfusion was performed in the majority of patients who underwent surgeries at Fuwai Hospital. The thresholds of blood transfusion were rigorous, and a few myocardial ischemia events were observed during the OPCAB surgery, such as new regional wall motion abnormalities, malignant arrhythmia, permanent ST segment elevation, or unexplained cardiac output reduction.

There is no consistency or guidelines with regard to the intraoperative anticoagulation and reversal strategy during OPCAB. The heparin dosage ranged from 70 to 500 U/kg and the trend dosage was 150 IU/kg with goal ACT of 200–450 s in most American and European cardiac surgery centers. Heterogeneity also existed in reversal strategy ranging from full-dose protamine to no reversal of anticoagulation (30). The loading dose of heparin before coronary anastomosis is 200 IU/kg in majority of our series, and the full-dose protamine for heparin reversal is frequently performed at the end of the coronary anastomosis procedure, indicating surgeons' concern about intraoperative bleeding and the confidence of the graft patency. A similar result was discovered in a survey of 750 cardiothoracic surgeons from 16 European countries (31).

### Inotropic and vasoactive agent

Pharmacological management plays an important role during the surgery. Nitroglycerin, as a nitric oxide donor for vasodilation, has remarkable beneficial effects on microcirculation in patients with heart failure (32). Reperfusion-related myocardial suppression constantly occurred after revascularization, and dopamine is an inotropic and vasoactive agent that is used to treat low blood pressure, a low heart rate, and cardiac arrest (33). Hence, continuous infusions of nitroglycerin and/or low-dose dopamine were the basic treatments during the periods of the operation room and the intensive care unit. Milrinone is a phosphodiesterase 3 inhibitor that increases cardiac inotropy, lusitropy, and peripheral vasodilatation (34). It also improves hemodynamic parameters and increases the blood flow of the grafted internal mammary arteries as well as the middle cerebral arteries during the surgery. Perioperative continuous infusion of milrinone is effective in lowering the incidence of myocardial ischemia and myocardial infarction in patients after the OPCAB surgery (35).

β-blockers have been proven to reduce the morbidity of postoperative atrial fibrillation and ventricular arrhythmia without increasing the incidences of hypotension or bradycardia in patients undergoing coronary arterial surgery (36). In the patients of this study, atenolol was frequently applied with bolus fashion, which can reduce the HR without obvious negative inotropic action.

Nicardipine is a highly selective and short-acting calcium-channel blocker. It has a strong vasodilation activity, including in coronary arteries, which approved for the treatment of chronic stable angina and hypertension without causing reflex tachycardia or negative inotropic action (37). Therefore, it is safe to use nicardipine in hemodynamic regulation during the OPCAB surgery.

Methoxamine is a highly selective α1 receptor agonist that raises blood pressure and causes the heart rate to slow down, which can increase coronary blood flow (38), so it is beneficial to alleviate myocardial ischemia due to hypotension and tachycardia. Therefore, methoxamine is frequently applied during the coronary anastomosis procedure at Fuwai Hospital.

This study has several limitations. First, this retrospective and single-center study did not include a control group, and the lack of a randomized double-blind design resulted in bias in the research conclusions. Second, the sample size might be insufficient due to heterogeneity among different anesthesiologists, surgeons, and patients. Finally, the observational results did not include the long-term outcomes of the patients who underwent OPCAB surgery.

## Conclusions

The present study summarized the anesthesia practice for OPCAB patients at a large-volume cardiovascular center in China with respect to intraoperative monitoring, anesthetics, inotropic and vasoactive agents, patient blood management, etc.

## Data Availability

The datasets presented in this article are not readily available because of the privacy of individuals that participated in the study. Requests to access the datasets should be directed to correspondent author.
