# Corrigendum: Anesthesia management of patients undergoing off-pump coronary artery bypass grafting: A retrospective study of single center

**DOI:** 10.3389/fsurg.2023.1160811

**Published:** 2023-03-08

**Authors:** Yong Lin, Tao Sun, Ning-ning Cheng, Jing-jing Liu, Li-xian He, Li-hong Wang, Xian-wen Liu, Mei-fang Chen, Liang-wan Chen, Yun-tai Yao, the Evidence in Cardiovascular Anesthesia (EICA) Group

**Affiliations:** ^1^ Department of Anesthesiology, Fuwai Hospital, National Center for Cardiovascular Diseases, Peking Union Medical College and Chinese Academy of Medical Sciences, Beijing, China; ^2^ Department of Cardiovascular Surgery, Fujian Medical University Union Hospital, Fuzhou, China; ^3^ Department of Anesthesiology, The Affiliated Hospital of Inner Mongolia Medical University, Huhhot, China; ^4^ Department of Anesthesiology, Binzhou People’s Hospital, Binzhou, China; ^5^ Department of Anesthesiology, The First Affiliated Hospital of Xinxiang Medical College, Xinxiang, China; ^6^ Department of Anesthesiology, Chuiyangliu Hospital of Tsinghua University, Beijing, China; ^7^ Department of Anesthesiology, Liaocheng People’s Hospital, Liaocheng, China

**Keywords:** coronary artery bypass, anesthesia management, single center, retrospective, hemodynamics

A Corrigendum on Anesthesia management of patients undergoing off-pump coronary artery bypass grafting: A retrospective study of single center By Lin Y, Sun T, Cheng N-n, Liu J-j, He L-x, Wang L-h, Liu X-w, Chen M-f, Chen L-w, Yao Y-t and the Evidence in Cardiovascular Anesthesia EICA) Group. (2023) Front. Surg. 9:1067750. doi: 10.3389/fsurg.2022.1067750

**Incorrect Correspondence Email**

In the published article, there was an error in correspondence email. Instead of “yuntaiyao666@126.com”, it should be “yuntaiyao@126.com”.

The authors apologize for this error and state that this does not change the scientific conclusions of the article in any way. The original article has been updated.

